# Human Papillomavirus Infection and Cervical Dysplasia in a Subset of Arab American Women

**DOI:** 10.1089/whr.2020.0129

**Published:** 2021-07-16

**Authors:** Anita Shallal, Evi Abada, Ziad Fehmi, Saivaishnavi Kamatham, Joseph Trak, Omar Fehmi, Andrew Toma, Sarah Farooqi, Hyejeong Jang, Seongho Kim, Sudeshna Bandyopadhyay, Marcus Zervos, Rouba Ali-Fehmi

**Affiliations:** ^1^ Division of Infectious Disease, Henry Ford Hospital, Detroit, Michigan, USA.; ^2^ Department of Pathology, Wayne State University School of Medicine/Detroit Medical Center, Detroit, Michigan, USA.; ^3^ University of Michigan, Ann Arbor, Michigan, USA.; ^4^ Detroit Medical Center/Wayne State University, Detroit, Michigan, USA.; ^5^ Oakland University, Rochester, Michigan, USA.; ^6^ Department of Oncology, Biostatistics Core, Karmanos Cancer Institute, Wayne State University School of Medicine, Detroit, Michigan, USA.

**Keywords:** HPV, Arab American women, cervical cancer screening, Pap smear, cervical dysplasia

## Abstract

***Background:*** With limited health data on Arab Americans (AAs), we sought to describe the health-seeking behaviors, prevalence of abnormal cervical cytology and high-risk human papillomavirus (HPV) serotypes, and the relationship with socioeconomic factors among a subset of AA women.

***Methods:*** Retrospective observational cohort study of women undergoing routine cancer screening at the Arab-American Center for Economic and Social Services clinic. Data collected included demographics, tobacco use, gross monthly income, prior Papanicolaou (Pap) smear history, and results of cervical cytology and high-risk HPV testing.

***Results:*** Of 430 women, 74 (17%) reported that they had never had a Pap smear. Three hundred eighty-eight (90%) women had cervical cytology interpreted as “negative for intraepithelial lesion,” the remaining 42 (10%) women had abnormal results. Thirteen (3%) women reported prior abnormal Pap smear, which was significantly associated with additional abnormal Pap smear on multivariable analyses (odds ratio 65.46; 95% confidence interval [CI] 17.01–338.62; *p* < 0.001). One hundred twenty-five (29%) women were tested for high-risk HPV serotypes; 106 (91%) had negative results, 4 (3%) were positive for HPV-16, 7 (6%) were positive for other high-risk serotypes, and 8 results were not recorded. A negative HPV screen was significantly associated with a negative Pap smear (Fisher's exact test *p* = 0.006). There was no significant association between abnormal cervical cytology and evaluated socioeconomic factors.

***Conclusions:*** Additional population based-studies to determine cervical dysplasia/cancer and HPV prevalence in women of Middle Eastern descent are needed.

## Background

It is estimated that cervical cancer is the second most common cause of cancer among women worldwide, and 80% of cases occur in developing countries.^1–3^ Persistent infection with human papillomavirus (HPV), a sexually transmitted infection, is known to be the primary cause of cervical cancer. Majority of cases are squamous cell carcinoma, followed by adenocarcinomas. The Papanicolaou (Pap) smear, a screening examination that detects abnormal cells in the cervix, is the single most effective tool available for early diagnosis and treatment of cervical cancer. The test can also be used to assess for the presence of high-risk oncogenic HPV strains (in particular, strains 16 and 18).^4^

Strong evidence supports the benefits of early detection in substantially reducing cervical cancer incidence and mortality. Therefore, deaths from cervical cancer can be used as a marker of health care inequity and inability to access health care. Despite the effectiveness of these screening strategies, significant racial and ethnic disparities in cervical cancer screening and detection still exists worldwide. These disparities are related to multiple intersecting factors, including race, ethnicity, culture, socioeconomic status, immigration status, religion, and nativity (country of citizenship at birth), which may influence an individual's ability to receive preventative care.^5^

In Middle Eastern countries, the Pap smear is less utilized, and organized screening programs for cervical cancer is limited.^6^ This results in a large percentage of women who are under or never screened.^7^ As a result, mortality rates for cervical cancer in the Middle East are disproportionately high when compared with developed countries, and the majority of cases are diagnosed at later stages.^7^ The mortality rates reflect barriers to assessing regular screening, including lack of health insurance, length of stay in the United States, lack of transportation or childcare, as well as costs of health care.^7^ Personal factors such as language barriers, lack of knowledge about importance of screening, as well as fear of pain or embarrassment from a male provider may also result in reduced screening.^3,8^ Cultural and religious beliefs are also barriers to screening, as one study revealed that single Arab women may not seek out gynecologic examinations or HPV vaccination due to fear of stigmatization.^5^

In light of the aforementioned, data on cervical cancer screening for Arab American (AA) women are significantly limited. The Arab-American Center for Economic and Social Services (ACCESS) clinic is the largest and most comprehensive Arab community-based health and mental health center in North America and is considered a “one stop service center” for medical, public health, mental health, and environmental programs for this particular community.^9^ ACCESS provides culturally sensitive and community-centered health care services to AA in Southeast Michigan. The aim of this study was to evaluate the health-seeking behaviors surrounding Pap smears, describe the prevalence of abnormal cervical cytology and high-risk HPV serotypes, and finally, to correlate the relationship between abnormal cervical cytology results and socioeconomic factors in this subset of AA women who received care at the ACCESS clinic.

## Methods

This retrospective observational cohort study was approved by the Wayne State University and the Henry Ford Health System Institutional Review Boards. We reviewed clinical records of AA women who presented for routine cancer screening as part of the Breast and Cervical Cancer Control Program (BCCCP) at the ACCESS Clinic in southeast Michigan. AA women include women with origins from the “Arab World” who have immigrated to and now reside in the United States. The “Arab World” includes 22 countries namely, Algeria, Bahrain, the Comoros Islands, Djibouti, Egypt, Iraq, Jordan, Kuwait, Lebanon, Libya, Morocco, Mauritania, Oman, Palestine, Qatar, Saudi Arabia, Somalia, Sudan, Syria, Tunisia, the United Arab Emirates, and Yemen. Medical records for these women were reviewed for information, including age, date of birth, gross monthly income, history of tobacco use, prior Pap smear history, including those who declined a Pap smear test, cervical cytology results, and high-risk HPV testing results. Gross monthly incomes were stratified into those earning <$2000 U.S. dollars (USD) and ≥$2000 USD per month. AA women who presented for care at the ACCESS clinic between 2003 and 2019 and who were between the ages of 21 and 65 years at the time of their visit were included in the study. Women who had a prior hysterectomy with removal of the cervix were excluded. All collected data were deidentified. In instances where cytology was performed, results were documented as unsatisfactory sample, negative for intraepithelial lesion, atypical cells of undetermined significance, atypical squamous cells cannot rule out high-grade squamous intraepithelial lesion, low-grade squamous intraepithelial lesion, high-grade squamous intraepithelial lesion, and atypical glandular cells. In instances where high-risk HPV testing was performed, negative results were documented as negative, and positive results were documented as positive for HPV 16, 18, or other high-risk strains (31, 33, 35, 39, 45, 51, 52, 56, 58, 59, 66, and 68) or unavailable if serotype testing result was not recorded.

### Statistical analysis

Patient characteristics were summarized by count and percentage for categorical variables and median and range for continuous variables. Fisher's exact tests and Wilcoxon rank-sum tests were used to compare categorical and continuous variables, respectively, between groups. Univariable and multivariable logistic regression analyses were performed to assess associations between five patient baseline characteristics (age, monthly income, tobacco use, never had Pap, and prior abnormal Pap) and cervical cytology (abnormal vs. normal, with normal as reference). In particular, Firth logistic regression models were used to reduce bias in maximum likelihood estimation caused by rare events. The association between cervical cytology and high-risk HPV was assessed by Fisher's exact tests.

## Results

A total of 464 charts from the BCCCP program were reviewed. Thirty-four cases with unavailable cervical cytology results were excluded from the analysis. Four hundred thirty women were included in the study. All women were uninsured and had immigrated from Middle Eastern countries, including Yemen and Lebanon. The average age of the women was 49 years (range 23–65). Women with abnormal^a^ Pap smears were significantly more likely to be younger in age (Wilcoxon rank-sum test *p* = 0.022). A summary of patient characteristics is presented in Table 1. Seventy-four (17%) women reported that they had never had a prior Pap smear. However, not having a previous Pap smear had no association with having an abnormal Pap smear at the BCCCP on univariable (odds ratio [OR] = 0.66; 95% confidence interval [CI] 0.23–1.59; *p* = 0.377) and multivariable (OR = 1.81; 95% CI 0.48–5.71; *p* = 0.355) analyses (Table 2). A total of 388 (90%) women had cervical cytology interpreted as “negative for intraepithelial lesion” at the BCCP. The remaining 42 (10%) women had abnormal cervical cytology results. Thirteen (3%) women reported prior abnormal Pap smear results before coming to the BCCCP and this was statistically significant with having another abnormal Pap smear on both univariable (OR = 47.74; 95% CI 14.06–203.85; *p* < 0.001) and multivariable analyses (OR = 65.46; 95% CI 17.01–338.62; *p* < 0.001) (Table 2). One hundred twenty-five (29%) women were tested for high-risk HPV serotypes. Of these, 106 (91%) had negative results, 4 (3%) were positive for HPV 16, 7 (6%) were positive for other high-risk HPV serotypes, and 8 results were not recorded. Having a negative HPV screen was significantly associated with having a negative Pap smear (Fisher's exact test *p* = 0.006; Table 3). Three hundred twenty-one (75%) women had a gross monthly income <$2000 USD. Eighty-six (20%) women reported using tobacco. However, having a gross monthly income <$2000 USD (OR = 0.43; 95% CI 0.10–1.36; *p* = 0.162) and history of tobacco use (OR = 0.72; 95% CI 0.16–2.40; *p* = 0.611) had no association with having an abnormal cervical Pap smear result (Table 2).

**Table 1.** Patient Characteristics

Variable		Cervical cytology		*p*^b^
	All (n = 430)	Normal^a^ (*n* = 388)	Abnormal^b^ (*n* = 42)	
Age, years, median (range)	49 (23–65)	49 (23–65)	47 (30–59)	0.022
Monthly income, *n* (%)				0.340
<$2000	321 (75)	287 (74)	34 (81)	
≥$2000	105 (24)	98 (25)	7 (17)	
Unknown	4 (1)	3 (1)	1 (2)	
Tobacco use, *n* (%)				0.685
Yes	86 (20)	79 (20)	7 (17)	
No	323 (75)	290 (75)	33 (79)	
Unknown	21 (5)	19 (5)	2 (5)	
Never had Pap, *n* (%)				0.389
Yes	74 (17)	69 (18)	5 (12)	
No	295 (69)	264 (68)	31 (74)	
Unknown	61 (14)	55 (14)	6 (14)	
Prior abnormal Pap smear, *n* (%)				<0.001
Yes	13 (3)	3 (1)	10 (24)	
No	295 (69)	278 (72)	17 (40)	
Unknown	122 (28)	107 (28)	15 (36)	
HPV testing done, *n* (%)				<0.001
Yes	125 (29)	89 (23)	36 (86)	
No	263 (61)	259 (67)	4 (10)	
Unknown	42 (10)	40 (10)	2 (5)	

^a^ Negative for intraepithelial lesion.

^b^ ASC-US (atypical cells of undetermined significance), ASC-H (atypical squamous cells cannot rule out high-grade squamous intraepithelial lesion), LSIL (low-grade squamous intraepithelial lesion), HSIL (high-grade squamous intraepithelial lesion), and AGC (atypical glandular cells).

^c^ Fisher's exact test or Wilcoxon rank-sum test as appropriate.

HPV, human papillomavirus; Pap, Papanicolaou.

**Table 2.** Univariable and Multivariable Logistic Regression Analyses of Risk Factors Associated with Cervical Cytology (Abnormal vs. Normal, with Normal as Reference)

	Univariable^a^			Multivariable^a^		
	Event/n	OR (95% CI)	*p*	Event/n	OR (95% CI)	*p*
Age	42/430	0.96 (0.93–1.00)	0.050	25/296	0.94 (0.88–1.01)	0.104
Monthly income						
<$2000	34/321	Ref.		19/221	Ref.	
≥$2000	7/105	0.63 (0.26–1.37)	0.259	6/75	0.43 (0.10–1.36)	0.162
Tobacco use						
No	33/323	Ref.		22/231	Ref.	
Yes	7/86	0.82 (0.33–1.78)	0.629	3/65	0.72 (0.16–2.40)	0.611
Never had Pap						
No	31/295	Ref.		21/253	Ref.	
Yes	5/74	0.66 (0.23–1.59)	0.377	4/43	1.81 (0.48–5.71)	0.355
Prior abnormal Pap						
No	17/295	Ref.		15/283	Ref.	
Yes	10/13	47.74 (14.06–203.85)	<0.001	10/13	65.46 (17.01–338.62)	<0.001

Event/n, numbers of events (abnormal) and patients.

^a^ Univariable and multivariable Firth logistic regression analyses, respectively.

CI, confidence interval; OR, odds ratio; Ref., reference.

**Table 3.** Association Between Cervical Cytology and High-Risk Human Papillomavirus Among Patients Who Had Human Papillomavirus Testing

High risk HPV	Cervical cytology^a^				Total (*N* = 125)	*p*^b^
	NIL (*n* = 89)	ASC-US (*n* = 29)	ASC-H (*n* = 2)	LSIL (*n* = 5)		
Negative	81 (95%)	22 (85%)	1 (50%)	2 (50%)	106 (91%)	0.006
Positive for HPV 16	2 (2%)	1 (4%)	0 (0%)	1 (25%)	4 (3%)	
Positive for other high-risk HPV^c^	2 (2%)	3 (12%)	1 (50%)	1 (25%)	7 (6%)	
Unknown	4	3	0	1	8	

^a^ The Bethesda System, 2001.

^b^ Fisher's exact test.

^c^ Other serotypes: 31, 33, 35, 39, 45, 51, 52, 56, 58, 59, 66, and 68.

## Discussion

Population-based screening programs have resulted in minimizing mortality and morbidity from cervical cancer.^6^ AA are a rapidly growing ethnic minority in the United States with a growth rate of 72% from 2000 to 2010.^5^ They are also particularly understudied with regard to preventive health behaviors, in part because this population is classified as “White” by the U.S. government, leaving them overlooked, and rendering them effectively invisible as an ethnic minority, and also leading to a lack of research being conducted with this population.^5^ Recently emigrated AA women are vulnerably situated at an intersection of race, ethnicity, socioeconomic status, culture, and religion. These women, who were already less likely to undergo screening in their home country before immigrating to the United States,^7^ may continue to decline screening even when it is offered, particularly given the cultural stigma associated with sexually transmitted infections. This behavior is evident in our results, where 17% of the participants had never undergone a Pap smear, compared with ∼6.8% of U.S. women.^10^ A secondary data analysis of the 2000–2011 National Health Interview Survey (NHIS) compared cancer screening behaviors of U.S.-born and foreign-born (European and Arab countries) non-Hispanic white women. After adjusting for age, foreign-born AA women were significantly less likely (84%) to receive a Pap test in their lifetime than European (87%) and U.S. (95%) women (*p* ≤ 0.00).^11^

According to the Centers for Disease Control and Prevention, risk factors for cervical cancer development include absence of cervical cancer screening, history of persistent infection with HPV, increasing number of lifetime sexual partners, early age of sexual activity, as well as history of other sexually transmitted infections, such as herpes simplex virus and *Chlamydia trachomatis*.^4^ In addition, a growing number of studies have shown that tobacco use may be an additional risk factor, with a dose–response relationship between tobacco use and cervical cancer.^12,13^ Although tobacco use is decreasing slowly in the United States, there remains a high prevalence of tobacco use within Middle Eastern countries.^14^ Within our study population, 20% of the women reported tobacco use. However, the use of tobacco was not significantly associated with abnormal cervical cytology in our study (*p* = 0.611; Table 2). In comparison, according to recent statistics, among factors contributing to cervical cancer, women in the United States had a smoking prevalence of 15.9%.^15^ Living in poverty is also a well-established risk factor for the development of cervical cancer.^16^ Our results indicate that most (75%) of the AA women in this study recorded making a gross monthly income <$2000 USD. However, a gross monthly income <$2000 USD was not significantly associated with abnormal cervical cytology (*p* = 0.162; Table 2). In contrast, data from the surveillance, epidemiology, and end results (SEER) study show that a lower socioeconomic status was associated with a risk of developing cervical cancer in U.S. women.^17^

Abnormal cervical Pap smears with an interpretation of atypical cells of undetermined significance or higher was seen in 42 (10%) of the women seen at the ACCESS clinic. Of these, high-grade squamous intraepithelial lesion/severe dysplasia and atypical glandular cells were seen in <1% of the women. No invasive carcinomas (squamous or glandular) were detected in this subset of women. High-risk HPV strains were detected in only 11 (9%) of the women with abnormal cervical cytology among those 125 women who were tested for HPV (Table 3). Of note is that HPV 18 serotypes were not detected in this subset of AA women who recently immigrated to the United States, whereas HPV 16 and other high-risk serotypes were variably detected. In comparison, results from a large U.S. screening population data indicates that HPV 16 genotype was more prevalent in women of all age groups.^18^ Serotype prevalence has been studied in other populations, from South America to East Asia.^19^ HPV genotype 16 and 18 have been identified as the predominant oncogenic types, causing ∼70% of all cervical cancer cases worldwide.^20^ However, there is limited to no data on seroprevalence across the Middle East. To our knowledge, this study is the first of its kind to look at seroprevalence among a subset of AA women who recently immigrated to the U.S. population-based studies to determine which serotypes are more common among this ethnicity are encouraged, as such knowledge may play a role in vaccine development and treatment. In addition, out of the 125 women from our study population tested for high-risk HPV serotypes, 106 (91%) had negative results. Nonetheless, having a negative HPV screen was significantly associated with having a negative Pap smear (Fisher's exact test *p* = 0.006; Table3). Our findings are consistent with what has been previously reported from a U.S.-based study, in which a negative HPV screen was correlated with a negative Pap smear result.^21^

Finally, given the prior literature on the rates of cervical cancer in Middle Eastern countries (particularly low-income countries), we anticipated identifying a higher rate of cervical dysplasia, as well as cervical cancer within our study cohort. However, this is not what was identified, and in fact, no cases of cervical cancer were diagnosed in our cohort. In comparison, the review by Islami et al. found that among non-Hispanic whites, the overall incidence rate of histologically confirmed cervical cancer decreased by 1.1% per year from 2003 to 2013; with rates stabilizing afterward (from 2013 to 2015).^22^ This finding of decreasing incidence in cervical cancer cases among non-Hispanic white women can be attributed to the effectiveness of cervical screening programs in the United States. We wish to note that cervical cancer screening is a well-established program within high-income countries such as the United States, where our study participants currently live.

### Study limitations

It is important to note that there are a number of limitations to this study. First, the retrospective nature of the study limits the available data and statistics. Next, as the medical records were all paper, issues encountered included lost sheets of paper without backup copies, illegible notes, or incomplete information. All attempts were made to collect the data without inference and to mark the data as incomplete or unavailable, where appropriate. Finally, due to the nature of the program at ACCESS, the charts that were reviewed only included a checklist of information limited to each woman's single cancer screening clinic visit, and thus we were unable to perform both prior record review and follow up.

## Conclusions

AA women are an understudied population and an increasing ethnic minority in the United States. Information regarding their health-seeking behaviors and specific disease prevalence are currently lacking. Therefore, health care providers should be aware of the challenges that face them, particularly with regard to cervical cancer screening and prevention. Additional studies targeting this demographic within the U.S. health system should be done to facilitate preventative health care and effective treatment strategies.

## Abbreviations Used

AA: Arab American
ACCESS: Arab-American Center for Economic and Social Services
AGC: atypical glandular cells
ASC-H: atypical squamous cells cannot rule out high-grade squamous intraepithelial lesion
ASC-US: atypical cells of undetermined significance
BCCCP: Breast and Cervical Cancer Control Program
CI: confidence interval
HPV: human papillomavirus
HSIL: high-grade squamous intraepithelial lesion
LSIL: low-grade squamous intraepithelial lesion
NHIS: National Health Interview Survey
NIL: negative for intraepithelial lesion
OR: odds ratio
Pap: Papanicolaou
SEER: surveillance, epidemiology, and end results
USD: U.S. dollars

## Notes

^a^ Abnormal cervical cytology (Pap smear) results recorded include; [atypical cells of undetermined significance, atypical squamous cells cannot rule out high grade squamous intraepithelial lesion, low grade squamous intraepithelial lesion, high grade squamous intraepithelial lesion, and atypical glandular cells.

## References

[1] World Health Organization. WHO Guidance Note: Comprehensive Cervical Cancer Prevention and Control: A Healthier Future for Girls and Women, 2013. Available at: https://apps.who.int/iris/bitstream/handle/10665/78128/9789241505147_eng.pdf;jsessionid=190CCA77B5D4F30AC224004BB7C460CF?sequence=3 Accessed 122, 2020

[2] Bruni L, Albero G, Serrano B, et al. Human Papillomavirus and Related Diseases in The World, 2016. Available at: https://www.hpvcentre.net/statistics/reports/XWX.pdf Accessed 122, 2020

[3] Jassim G, Obeid A, Al Nasheet HA. Knowledge, attitudes, and practices regarding cervical cancer and screening among women visiting primary care centres in Bahrain. BMC Public Health 2018;18:128 29325528 10.1186/s12889-018-5023-7 PMC5765703

[4] Centers for Disease Control and Prevention. Cervical cancer: What are the risk factors for cervical cancer? 2019. Available at: https://www.cdc.gov/cancer/cervical/basic_info/risk_factors.htm Accessed 122, 2020

[5] Abboud S, De Penning E, Brawner BM, Menon U, Glanz K, Sommers MS. Cervical cancer screening among Arab women in the United States: An integrative review. Oncol Nurs Forum 2017;44:E20–E33 27991600 10.1188/17.ONF.E20-E33 PMC5553625

[6] Ali S, Skirton H, Clark MT, Donaldson C. Integrative review of cervical cancer screening in Western Asian and Middle Eastern Arab countries. Nurs Health Sci 2017;19:414–426 29058371 10.1111/nhs.12374

[7] Sharma M, Seoud M, Kim JJ. Cost-effectiveness of increasing cervical cancer screening coverage in the Middle East: An example from Lebanon. Vaccine 2017;35:564–569 28017434 10.1016/j.vaccine.2016.12.015

[8] Coughlin SS, Uhler RJ. Breast and cancer screening practices among Hispanic women in the United States and Puerto Rico, 1998–1999. Prev Med 2002;34:242–251 11817921 10.1006/pmed.2001.0984

[9] Arab Community Center for Economic and Social Services. ACCESS website. Available at: http://www.accesscommunity.org/ Accessed 122, 2020

[10] Clark TC, Endeshaw M, Senkomago V, Saraiya M. QuickStats: Percentage of U.S. women aged 21–65 years who never had a Papanicolaou test (Pap test), by place of birth and length of residence in the United States—National Health Interview Survey, 2013 and 2015. MMWR Morb Mortal Wkly Rep 2017;66:346 28358794 10.15585/mmwr.mm6612a9 PMC5657958

[11] Dallo FJ, Booza J, Nguyen ND. Functional limitations and nativity status among older Arab, Asian, Black, Hispanic, and White Americans. J Immigr Minor Health 2015;17:535–542 24165988 10.1007/s10903-013-9943-0 PMC4004727

[12] IARC Working Group on the Evaluation of Carcinogenic Risks to Humans. Personal Habits and Indoor Combustions: A Review of Human Carcinogens, Vol. 100E, 2012. Available at: https://publications.iarc.fr/122 Accessed 122, 2020 PMC4781577 23193840

[13] Cogliano VJ, Baan R, Straif K, et al. Preventable exposures associated with human cancers. J Natl Cancer Inst 2011;103:1827–1839 22158127 10.1093/jnci/djr483 PMC3243677

[14] World Health Organization. Prevalence of tobacco smoking: Age-standardized prevalence of current tobacco smoking among persons aged 15 years and older (%), 2015: male, 2016. Available at: http://gamapserver.who.int/gho/interactive_charts/tobacco/use/atlas.html Accessed 122, 2020

[15] Monsonego J, Cox JT, Behrens C, et al. Prevalence of high-risk human papilloma virus genotypes and associated risk of cervical precancerous lesions in a large U.S. screening population: Data from the ATHENA trial. Gynecol Oncol 2015;137:47–54 25667973 10.1016/j.ygyno.2015.01.551

[16] Knaul FM, Rodriguez NM, Arreola-Ornelas HA, Olson JR. Cervical cancer: Lessons learned from neglected tropical diseases. Lancet Glob Health 2019;7:e299–e300 30728108 10.1016/S2214-109X(18)30533-3

[17] The Catalan Institute of Oncology (ICO) and the International Agency for Research on Cancer (IARC), Human Papillomavirus and Related Cancers Fact Sheet, 2018. Available at: https://hpvcentre.net/statistics/reports/USA_FS.pdf Accessed 329, 2021

[18] Clegg LX, Reichman ME, Miller BA, et al. Impact of socioeconomic status on cancer incidence and stage at diagnosis: Selected findings from the surveillance epidemiology, and end results: National Longitudinal Mortality Study. Cancer Causes Control 2009;20:417–435 19002764 10.1007/s10552-008-9256-0 PMC2711979

[19] HPV Information Centre. Statistics, 2020. Available at: https://hpvcentre.net/datastatistics.php Accessed 122, 2020

[20] Ali MAM, Bedair RN, Abd El Atti RM. Cervical high-risk human papillomavirus infection among women residing in the Gulf Cooperation Council countries: Prevalence, type-specific distribution, and correlation with cervical cytology. Cancer Cytopathol 2019;127:567–577 31390155 10.1002/cncy.22165

[21] Gage JC, Schiffman M, Katki HA, et al. Reassurance against future risk of precancer and cancer conferred by a negative human papillomavirus test. J Natl Cancer Inst 2014;106:dju153 25038467 10.1093/jnci/dju153 PMC4111283

[22] Islami F, Fedewa SA, Jemal A. Trends in cervical cancer incidence rates by age, race/ethnicity, histological subtype, and stage at diagnosis in the United States. Prev Med 2019;123:316–323 31002830 10.1016/j.ypmed.2019.04.010

